# An update on prevention of venous thromboembolism in hospitalized acutely ill medical patients

**DOI:** 10.1186/1477-9560-4-8

**Published:** 2006-07-03

**Authors:** Meyer Michel Samama, Franz-Xaver Kleber

**Affiliations:** 1 Départment d'Hématologie Biologique, Hôtel Dieu, Paris, France; 2 Charité Medical School, Unfallkrankenhaus Berlin Academic Teaching Hospital, Berlin, Germany

## Abstract

Both the recently updated consensus guidelines published by the American College of Chest Physicians, and the International Union of Angiology recommend thromboprophylaxis with either low-molecular-weight heparin (LMWH) or unfractionated heparin (UFH) in medical patients at risk of VTE. However, no guidance is given regarding the appropriate dosing regimens that should be used for thromboprophylaxis in this patient group. LMWH (enoxaparin and dalteparin) and UFH have been shown to be effective for thromboprophylaxis in at-risk hospitalized medical patients. Although LMWH once daily (o.d.) has been shown to be as effective as UFH three times daily (t.i.d.) for thromboprophylaxis in at-risk medical patients, there are no data to show that UFH twice daily (b.i.d) is as effective as either LMWH o.d. or UFH t.i.d. On the basis of currently available evidence, the LMWHs enoxaparin and dalteparin are more attractive alternatives to UFH for the prevention of VTE in hospitalized medical patients because of their convenient once-daily administration and better safety profile, demonstrated in terms of reduced bleeding, HIT, and other adverse events.

## Introduction

In the absence of thromboprophylaxis, the incidence of venous thromboembolism (VTE) ranges from 10–20% in general medical patients to 80% in trauma patients, spinal cord injury patients, and patients in the critical care unit [1,2]. Despite evidence from large, randomized clinical studies demonstrating the benefits of providing thromboprophylaxis for hospitalized medical patients at risk of VTE [3-5], thromboprophylaxis is not currently prescribed to the extent that might be expected in this patient population, leaving many patients exposed to significant risk of acute thrombotic complications and their long-term consequences [6-9].

Consensus guidelines published by the American College of Chest Physicians (ACCP) and the International Union of Angiology (IUA) recommend assessment of all hospitalized medical patients for the risk of VTE and the provision of appropriate thromboprophylaxis [1,2]. Furthermore, simple and clinically-relevant risk assessment models (RAMs) are available to facilitate VTE risk assessment [10,11]. A recently published evidence-based RAM, developed specifically for hospitalized medical patients, should provide additional guidance to physicians in this patients group [12]. This RAM integrates patient VTE risk level with appropriate thromboprophylactic strategies in the form of a management algorithm. Computerised reminders have also been shown to be valuable for improving prophylaxis prescribing rates [13], and an electronic risk assessment tool has recently been developed for use in medical patients as well as surgical patients [14].

The recently updated ACCP consensus guidelines give a grade 1A recommendation for thromboprophylaxis using either low-molecular-weight heparin (LMWH) or low-dose unfractionated heparin (UFH) in medical patients with congestive heart failure (CHF) or severe respiratory disease, or in medical patients who are confined to bed and have one or more risk factors for VTE, such as active malignancy, acute neurological disease, inflammatory bowel disease, previous VTE, or sepsis [1]. LMWHs, unlike UFH, have greater bioavailability and consistent anticoagulation effects, and the benefit of once-daily dosing [15,16].

The aim of this manuscript is to review recent advances in thromboprophylaxis in hospitalized medical patients, and discuss them in light of the recently updated ACCP consensus guidelines.

### Heparin-based thromboprohylaxis

Several placebo-controlled studies have investigated the efficacy of thromboprophylaxis with UFH or LMWH in medical patients (Table 1) [3-5,17-26]. In general, low-dose UFH, given subcutaneously (s.c.) twice daily (b.i.d.) or three times daily (t.i.d.), is effective in the prevention of VTE [17-21]. Nevertheless, some studies did not show a reduction in overall mortality [22,23], which led some physicians to question the value of thromboprophylaxis in medical patients [25,27].

**Table 1:** Randomized studies comparing the efficacy of thromboprophylaxis using LMWH, UFH or fondaparinux with placebo or no thromboprophylaxis in medical patients.

**Study**	**Patients**	**Detection of VTE**	**Dose regimen**	**Efficacy (thromboprophylaxis vs placebo or no thromboprophylaxis)**
Gallus et al.1973 [17]	Suspected AMI (n = 78)	^125^I-fibrinogen scanning	UFH, 5000 IU s.c. t.i.d.	VTE: 2.6% vs 22.5% (*p *= 0.05)
Belch et al. 1981 [18]	HF and/or chest infection (n = 100)	^125^I-fibrinogen scanning	UFH, 5000 IU s.c. t.i.d.	VTE: 4% vs 26% (*p *< 0.01)
Halkin et al. 1982 [19]	General medical (n = 1358)	Mortality study	UFH, 5000 IU s.c. b.i.d.	Mortality: 7.8% vs 10.9% (*p *< 0.05)
Cade, 1982 [20]	1) Medical (n = 131) 2) Critically ill (n = 119)	^125^I-fibrinogen scanning	UFH, 5000 IU s.c. b.i.d.	1) VTE: 2% vs 10% (*p *= NS) 2) VTE: 13% vs 29% (*p *< 0.05)
Ibarra-Perez et al. 1988 [21]	Pulmonary disease, >40 years (n = 85)	^125^I-fibrinogen scanning, plus contrast venography	UFH, 5000 IU s.c. b.i.d.	VTE: 2.6% vs 26.1% (*p *< 0.0022)
Gårdlund, 1996 [22]	Infectious disease (n = 19,751)	Autopsy-verified pulmonary embolism	UFH, 5000 IU s.c. b.i.d.	Mortality: 5.3% vs 5.6% (*p *= NS)
International Stroke Trial, 1997 [23]	Suspected acute ischaemic stroke (n = 19,435)	Mortality study	UFH, 5000 or 12,500 IU s.c. b.i.d.	14-day mortality: 9% vs 9.3% (*p *= NS) 6-month mortality: 22.5% vs 21.5% (*p *= NS)
Dahan et al. 1986 [24]	Medical, >65 years (n = 270)	^125^I-fibrinogen scanning	Enoxaparin 60 mg s.c. o.d.	VTE: 3% vs 9% (*p *= 0.03)
Bergmann and Caulin, 1996 [25]	Medical (n = 2472)	Mortality study; autopsy-confirmed pulmonary embolism	Nadroparin 7500 antiXa IU s.c. o.d.	Mortality: 10.1% vs 10.3% (*p *= NS)
Samama et al. 1999 [3]	Acutely ill medical (n = 1102)	Bilateral venography or duplex ultrasonography	Enoxaparin 1) 20 mg s.c. o.d. 2) 40 mg s.c. o.d.	1) VTE: 15.0% vs 14.9% (*p *= NS) 2) VTE: 5.5% vs 14.9% (*p *< 0.001)
Fraisse et al. 2000 [26]	Chronic obstructive pulmonary disease (n = 223)	Bilateral venography	Nadroparin 3800 or 5700 IU antiXa s.c. o.d.^1^	VTE: 15.5% vs 28.2% (*p *= 0.045)
Leizorovicz et al. 2004 [4]	Acutely ill medical (n = 3706)	Compression ultrasonography	Dalteparin 5000 IU s.c. o.d.	VTE: 2.8% vs 5.0% (*p *= 0.0015)
Cohen et al. 2006 [5]	Acutely ill elderly medical (n = 849)	Bilateral venography	Fondaparinux, 2.5 mg s.c. o.d.	VTE: 5.6% vs 10.5% (*p *= 0.029)

^1^Dose adjusted based on patients' body weight. Patients in the range 45–70 kg received 3800 antiXa IU and patients in the range 71–110 kg received 5700 antiXa IU (i.e. 0.4 ml or 0.6 ml of a 9500 antiXa IU/ml concentrated solution of nadroparin, respectively).

AMI, acute myocardial infarction; b.i.d., twice daily; HF, heart failure; LMWH, low-molecular-weight heparin; o.d., once daily; s.c. subcutaneously; t.i.d., three times daily; UFH, unfractionated heparin; VTE, venous thromboembolism.

The efficacy of LMWH thromboprophylaxis in hospitalized medical patients has been investigated in several large, randomized, placebo-controlled clinical studies (Table 1). Compared with placebo, thromboprophylaxis with LMWH reduces the risk of VTE by 45–66% [3,4,24-26]. In the international Prophylaxis in Medical Patients with Enoxaparin (MEDENOX) study, two doses of enoxaparin (20 mg and 40 mg s.c. o.d.) were compared with placebo in acutely ill medical patients [3]. The MEDENOX study, in which deep-vein thromboses (DVT) were confirmed using venography, showed a significant reduction in the incidence of VTE when 40 mg enoxaparin was used compared with placebo for 6–14 days (relative risk, 0.37; 97.6% confidence interval (CI), 0.22–0.63; *p *< 0.001), but not when 20 mg enoxaparin was used (Table 1). The benefit with 40 mg enoxaparin was maintained at 3-month follow-up (relative risk, 0.41; 95% CI, 0.25–0.68; *p *< 0.001). Moreover, Kaplan-Meier plots of the probability of 3-month survival rates suggest that the overall mortality was lower in patients receiving 40 mg enoxaparin than those receiving placebo, although differences in mortality rates between the three groups did not reach statistical significance. A retrospective multihospital analysis of data from hospitalized acutely ill medical patients in the USA also showed that thromboprophylaxis with enoxaparin resulted in a significant reduction in the incidence of VTE compared with no thromboprophylaxis (1.9% vs 6.2%, *p *= 0.023) [28].

The Prospective Evaluation of Dalteparin Efficacy for Prevention of VTE in Immobilized Patients Trial (PREVENT) later confirmed the benefits of LMWH prophylaxis in hospitalized medical patients. This study, which enrolled a patient population with a slightly lower thrombotic risk than those in MEDENOX and used compression ultrasound to confirm DVT, showed that a high thromboprophylactic dose of 5,000 IU dalteparin significantly reduced the incidence of VTE in acutely ill medical patients compared with placebo (relative risk, 0.55; 95% CI, 0.38–0.80; *p *= 0.0015) [4]. However, in contrast to the findings of the MEDENOX study [3], the reduced incidence of VTE was not statistically significant at the 3-month follow-up (relative risk, 0.70; 95% CI 0.36–1.35) and a trend towards reduced mortality was not observed in the dalteparin group compared with the placebo group [4]. It is difficult to conclude whether these differences between the findings of the MEDENOX and PREVENT studies were a result of different pharmacological properties of the two LMWHs [29] or differences in the methodology and patient characteristics of the two studies.

Several randomized studies have compared the efficacy of LMWH and UFH as thromboprophylaxis in hospitalized medical patients at risk of VTE (Table 2) [30-34]. These studies have all reported that LMWH is at least as effective as UFH in reducing the risk of VTE. Similarly, a meta-analysis of randomized studies of thromboprophylaxis with LMWH or UFH in medical patients confirmed that both LMWH and UFH are effective in reducing the incidence of DVT in these patients compared with placebo or no thromboprophylaxis (relative risk, 0.44; 95% CI, 0.29–0.64; *p *< 0.001) [35]. This meta-analysis also demonstrated that the rate of pulmonary embolism (PE) was significantly reduced by LMWH or UFH compared with placebo (relative risk, 0.48; 95% CI, 0.34–0.68; *p *< 0.001) [35]. No significant differences in the incidences of DVT or PE in patients receiving LMWH compared with UFH were observed (relative risk for DVT, 0.83; 95% CI, 0.56–1.24; *p *= 0.37; relative risk for PE, 0.74; 95% CI, 0.79–1.88; *p *= 0.52). It should be noted that this meta-analysis did not include data from the MEDENOX and PREVENT studies, and it also has some limitations in terms of differences in study designs and the small size of some of the studies included. Nevertheless, it does provide an important insight into the impact of heparin therapy on VTE risk in medical patients. Notably, a recent *post-hoc *analysis demonstrated that asymptomatic proximal DVT, which was more commonly diagnosed than symptomatic DVT in acutely ill medical patients in the major clinical studies mentioned above [3,4], is associated with increased mortality [36]. This adds weight to the clinical opinion that asymptomatic proximal DVT is a relevant surrogate endpoint for symptomatic thromboembolic disease.

**Table 2:** Randomized studies of thromboprophylaxis with LMWH compared with UFH in medical patients.

**Study**	**Patients**	**LMWH**	**UFH**	LMWH as effective as UFH?*
Harenberg et al. 1990 [30]	Hospitalised, bedridden medical (n = 166)	Dalteparin 1500 aPTT units s.c. o.d.	5000 IU s.c. t.i.d.	Yes
Bergmann and Neuhart, 1996 [31]	Elderly, bedridden, acutely ill medical (n = 442)	Enoxaparin 20 mg s.c. o.d.	5000 IU s.c. b.i.d.	Yes
Lechler et al. 1996 [32]	Medical (n = 959)	Enoxaparin 40 mg s.c. o.d.	5000 IU s.c. t.i.d.	Yes
Harenberg et al. 1996 [33]	Hospitalised, bedridden medical (n = 1968)	Nadroparin 36 mg s.c. o.d.	5000 IU s.c. t.i.d.	Yes
Kleber et al. 2003 [34]	Severe respiratory disease or acute heart failure (n = 665)	Enoxaparin 40 mg s.c. o.d.	5000 IU s.c. t.i.d.	Yes

*Efficacy defined as the incidence of objectively confirmed VTE in all studies.

aPTT, activated partial thromboplastin time; b.i.d., twice daily; LMWH, low-molecular-weight heparin; o.d., once daily; s.c., subcutaneously; t.i.d., three times daily; UFH, unfractionated heparin; VTE, venous thromboembolism.

### Thromboprophylactic regimens for medical patients

Although the recently updated ACCP consensus guidelines recommend thromboprophylaxis with either LMWH or UFH for at-risk medical patients, no recommendations are given regarding appropriate dosing regimens [1]. The ACCP guidelines' grade 1A recommendations are based in part on randomized clinical studies of thromboprophylaxis with LMWH given s.c. o.d. versus UFH given s.c. t.i.d. in medical patients. These studies showed that LMWH s.c. o.d. and UFH s.c. t.i.d. have similar efficacy and, therefore, both received a grade 1A recommendation. Despite these evidence-based guidelines, many physicians still use b.i.d. dosing for UFH in medical patients. This is probably due to data from earlier placebo-controlled clinical studies of UFH (Table 1), which showed that b.i.d. dosing with UFH was effective in reducing the risk of VTE in these patients [19-21].

When comparing UFH and LMWH, similar efficacy in the prevention of VTE in medical patients has only been observed when UFH t.i.d. was compared with LMWH o.d. (Table 2). One study did show that LMWH o.d. was as effective as UFH b.i.d. at preventing VTE in medical patients [31]. However, the dose of LMWH used was 20 mg enoxaparin s.c. o.d., a dose which the MEDENOX study showed to be ineffective for preventing VTE in acutely ill medical patients [3].

These findings were confirmed in a recently published systematic review of published randomized clinical trials evaluating the efficacy of UFH 5000 IU b.i.d., compared with LMWH and UFH 5000 IU t.i.d. [37]. Although UFH b.i.d. reduced the relative risk of VTE compared with placebo (relative risk 0.40, 95% CI 0.22–0.73), it was less effective than UFH t.i.d, versus placebo (relative risk 0.28, 95% CI 0.21–0.38). In studies comparing UFH 5000 IU t.i.d. with enoxaparin 40 mg o.d., enoxaparin was more effective at reducing the risk of VTE (relative risk 1.42; 95% CI 0.99–2.05).

**Figure 1:**
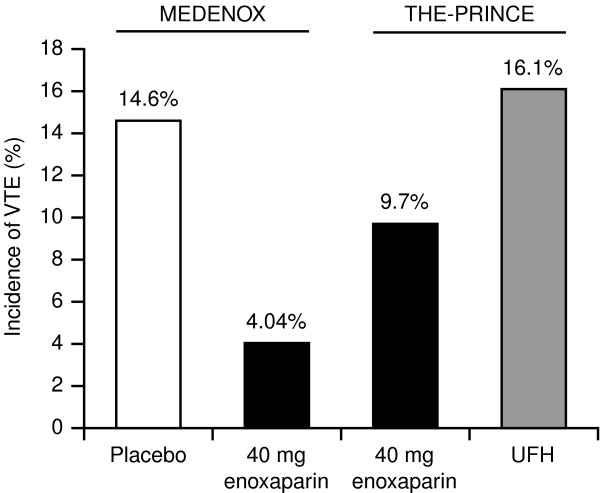
Incidence of VTE in congestive heart failure patients from the MEDENOX and THE-PRINCE studies [3, 34]. *P*-values were calculated using Fisher's exact test comparing pooled enoxaparin 40 mg data from the MEDENOX and THE-PRINCE studies with the placebo data from MEDENOX and the UFH data from THE-PRINCE. UFH, unfractionated heparin; VTE, venous thromboembolism.

In light of this, it is noteworthy that there are data to suggest better efficacy with enoxaparin than with UFH given on a t.i.d. basis to patients with congestive heart failure (CHF). When comparing enoxaparin 40 mg o.d. with UFH in patients with CHF in THE-PRINCE study, enoxaparin 40 mg o.d. showed a trend towards better efficacy than UFH, t.i.d. (Figure 1). Venous thromboembolic events occurred in 9.7% of the patients with CHF receiving enoxaparin 40 mg compared with 16.1% of those receiving UFH, although the patient numbers were too small to show a statistically significant difference between groups [34]. In patients with CHF in the MEDENOX study [3], which did not have a UFH arm, 40 mg enoxaparin resulted in a significant reduction in the incidence of VTE compared with placebo (relative risk, 0.29; 95% CI, 0.10–0.84; Figure 1) [38]. The lower incidence of VTE in CHF patients receiving enoxaparin 40 mg compared with either placebo or UFH warrants further investigation in a randomized clinical trial with sufficient power to show superior efficacy of enoxaparin, 40 mg o.d., compared with UFH, t.i.d., in CHF patients. It should also be noted that while the definitions of CHF patients were the same in these two studies, more patients presented with New York Heart Association (NYHA) class III-IV or IV in THE-PRINCE study than in the MEDENOX study (64.0% vs 20.2%). In addition, patients in THE-PRINCE study had more additional thrombotic risk factors, such as obesity (30% vs 20%) and pre-existing chronic venous disease (40% vs 25%). This may explain the higher incidence of VTE in patients with CHF in the enoxaparin, 40 mg o.d., arm of THE-PRINCE study (9.7%), compared with the enoxaparin 40 mg o.d. arm of the MEDENOX study (4.0%).

In summary, only the t.i.d. regimen of UFH is supported by similar clinical outcomes compared with LMWH o.d.. Therefore, only UFH t.i.d. amd LMWH o.d. (enoxaparin or dalteparin) and can be recommended for use as thromboprophylaxis in hospitalized medical patients at risk of VTE.

### Thromboprophylaxis with other anticoagulants

Although most studies on thromboprophylaxis in acutely ill medical patients have investigated the efficacy of LMWHs, a placebo-controlled trial of the synthetic factor Xa inhibitor fondaparinux (the Arixtra for Thromboembolism Prevention in a Medical Indications Study [ARTEMIS]) has recently been published [5]. The ARTEMIS study, which used venography to confirm DVT, showed a significant reduction in the incidence of VTE in elderly acutely ill medical patients receiving fondaparinux, 2.5 mg o.d., compared with placebo (5.6% vs. 10.5%, respectively, *p *= 0.029; relative risk reduction 46.7%; 95% CI 7.7%-69.3%) (Table 1) [5]. There was also a significant reduction in the incidence of symptomatic fatal or non-fatal pulmonary embolism in the fondaparinux group compared with the placebo group (1% vs. 3%, *p *= 0.029). Patient inclusion criteria in ARTEMIS differed from those used in the MEDENOX and PREVENT studies: patients were older (>60 years compared to >40 years) and were immobilised for longer (>4 days compared with ≤ 3 days) [3-5]. This may explain the high mortality rate in the placebo group of the ARTEMIS study; almost twice as many deaths occurred in the placebo arm compared with the fondaparinux arm (6.0% vs 3.3%, *p *= 0.06) [5]. Considering that the efficacy of thromboprophylaxis with LMWH or UFH is now firmly established in medical patients at risk of VTE, and that thromboprophylaxis with LMWH or low-dose UFH has a grade 1A recommendation from the ACCP consensus guidelines, the ethics of including a placebo arm in a study of this patient group is now questionable. Currently, only UFH, enoxaparin and dalteparin are approved for use in the prevention of VTE in medical patients. Further studies comparing fondaparinux with UFH or LMWH are awaited.

### Safety of thromboprophylaxis

Low-molecular-weight heparin o.d. has similar efficacy to UFH t.i.d. when used as thromboprophylaxis. However, several randomized clinical studies have shown that LMWH has a better safety profile than UFH (Table 3) [30-34]. In the Thromboembolism Prophylaxis in Internal Medicine with Enoxaparin (THE PRIME) study, bleeding events were similar between both treatment groups, but injection-site haematomas >5 cm were more frequently reported in patients who received UFH compared with those who received enoxaparin 40 mg o.d. (10.8% vs 4.6%, *p *< 0.001) [32]. Several other studies have reported similar results (Table 3).

**Table 3:** Safety of thromboprophylaxis with LMWH compared with UFH in medical patients.

**Study**	**Patients**	**LMWH**	**UFH**	**Safety**
Harenberg et al.1990 [30]	166	Dalteparin 1500 aPTT units s.c. o.d.	5000 IU s.c. t.i.d.	Similar incidence of adverse events in both groups. Haematomas were significantly smaller in diameter in the LMWH group
Bergmann and Neuhart, 1996 [31]	439	Enoxaparin 20 mg s.c. o.d.	5000 IU s.c. b.i.d.	Similar incidence of adverse events in both groups
Lechler et al. 1996 [32]	959	Enoxaparin 40 mg s.c. o.d.	5000 IU s.c. t.i.d.	Fewer major bleeding complications and significantly fewer injection-site haematomas (>5 cm diameter) in the enoxaparin group
Harenberg et al. 1996 [33]	1,968	Nadroparin 36 mg s.c. o.d.	5000 IU s.c. t.i.d.	Major bleeding was rare. Local haematomas (>2.5 cm diameter), local erythema and local allergic reactions were more frequent in the UFH group.
Kleber et al. 2003 [34]	665	Enoxaparin 40 mg s.c. o.d.	5000 IU s.c. t.i.d.	Significantly fewer adverse events in the enoxaparin group

aPTT, activated partial thromboplastin time; b.i.d., twice daily; LMWH, low-molecular-weight heparin; o.d., once daily; s.c., subcutaneously; t.i.d., three times daily; UFH, unfractionated heparin.

In the previously described meta-analysis of randomized studies that showed equivalent thromboprophylactic efficacy when directly comparing LMWH and UFH [35], use of LMWH also reduced the relative risk of major haemorrhage by 52% compared with UFH (relative risk, 0.48; 95% CI, 0.23–1.00; *p *= 0.049) [35]. Another, more recent, analysis pooled safety data from 2,346 patients from the MEDENOX, THE-PRINCE, and THE PRIME studies to determine the risk of haemorrhage following thromboprophylaxis with UFH or enoxaparin [39]. While the incidence of major haemorrhage was similar in patients given enoxaparin, UFH, or placebo, the incidence of minor bleeding associated with UFH was significantly greater than that associated with enoxaparin (relative risk, 1.7; 95% CI, 1.3–2.2; *p *= 0.0001).

The ARTEMIS study [5] reported a low risk of bleeding complications when elderly acutely ill medical patients were given thromboprophylaxis with fondaparinux 2.5 mg o.d.: major bleeding occurred in one patient in the fondaparinux group (0.2%) and one in the placebo group (0.2%). Minor bleeding occurred in 11 patients (2.6%) in the fondaparinux group and four in the placebo group (1.0%) [5].

The risk of heparin-induced thrombocytopenia (HIT) should also be considered when providing patients with thromboprophylaxis. The risk of HIT has been shown to be significantly lower in surgical patients receiving LMWH prophylaxis than in those receiving UFH [40]. Recent studies, however, suggest that the incidence of HIT in UFH-treated medical patients may be lower than in surgical patients [31,41]. Nevertheless, HIT remains an important medical issue because of the associated risk of thromboembolic events. A recent prospective cohort study reported that the incidence of HIT in hospitalized medical patients receiving subcutaneous UFH was less than 1%, but HIT was associated with a high incidence (60%) of thromboembolic events [41].

### Cost-effectiveness of thromboprophylaxis

Several economic studies have examined the cost-effectiveness of thromboprophylaxis in medical patients [42-45]. Three studies based on data from the MEDENOX study [3] showed that thromboprophylaxis with enoxaparin (40 mg s.c. o.d.) was cost-effective compared with placebo when examined from French, Canadian and Spanish cost perspectives [42-44]. A UK study of combined data from the MEDENOX study and a meta-analysis by Mismetti et al [35] showed that enoxaparin (40 mg s.c. o.d.) was associated with fewer VTE events and lower costs compared with no thromboprophylaxis [45] and was cost neutral compared with UFH.

## Conclusion

Evidence from clinical studies has shown the benefits of thromboprophylaxis with LMWH (enoxaparin and dalteparin) or UFH for the prevention of VTE in at-risk medical patients. Thromboprophylaxis with LMWH is as effective as UFH at preventing VTE in this group of patients, but has a significantly better safety profile. If UFH is used, physicians should choose a t.i.d. dose regimen, as there are no data to show that UFH b.i.d. is as effective as LMWH o.d. or UFH t.i.d. dosing. Recent data suggest that fondaparinux may be an effective and safe option in high-risk elderly medical patients, but further studies are awaited before general recommendations can be given regarding its use in all acutely ill medical patients. On the basis of currently available evidence, it appears that, while low-dose UFH is also recommended by the ACCP [1] although without specifying a dose regimen, the LMWHs enoxaparin and dalteparin may be more attractive alternatives for the prevention of VTE in medical patients because of their convenient once-daily administration and better safety profile, in terms of fewer bleeding complications, and a lower risk of HIT and other adverse events.

## Competing interests

MM Samama has periodically been an independent consultant for several pharmaceutical companies.

FX Kleber has no competing interests.

## Authors' contributions

Both authors had significant roles in conception, drafting and reviewing the manuscript, and they approved the final version.
